# Modified Chevrel Technique: A Lifesaver for Surgeons

**DOI:** 10.3390/medicina60081328

**Published:** 2024-08-16

**Authors:** Özcan Dere, Cenk Yazkan, Samet Şahin, Okay Nazlı, Önder Özcan

**Affiliations:** Department of Surgery, Faculty of Medicine, Muğla Sitki Koçman University, Mugla 48000, Turkey

**Keywords:** modified Chevrel technique, ventral hernia, onlay mesh hernia repair

## Abstract

*Background and Objectives:* Ventral hernias (VH) pose significant challenges for surgeons due to the risk of recurrence, complexities in aligning abdominal muscles, and selecting the most suitable layer for mesh augmentation. This study aims to evaluate the effectiveness of utilizing the anterior rectus fascia as a turnover flap in conjunction with onlay mesh reinforcement, a procedure known as the modified Chevrel technique (MCT). *Materials and Methods*: We conducted a retrospective analysis of patients who were operated on using MCT for abdominal hernias between January 2013 and December 2019. Data were extracted from our hospital’s electronic database. Recurrence rates, as well as the rates of surgical site occurrences (SSO), surgical site infections (SSI), and surgical site occurrences requiring procedural intervention (SSOPI), were analyzed based on patients’ comorbidities and demographic characteristics. *Results*: The median follow-up period was 42.9 months (range: 14–96), and the recurrence rate was 4% (n = 3). Among the recurrent cases, three patients had chronic obstructive pulmonary disease, representing a statistically significant association (*p* = 0.02). Although all patients with recurrence were obese, this association did not reach statistical significance (*p* > 0.05). The mean hospitalization duration was 17.6 days (range: 6–29). SSO, SSI, and SSOPI rates were 39 (52%), 12 (16%), and 32 (42%), respectively. *Conclusions*: Managing VH remains a surgical challenge, emphasizing the importance of achieving effective abdominal closure for both functional and cosmetic outcomes. MCT presents a relatively simple approach compared to techniques like transversus abdominis release (TAR) and anterior component separation (ACS), with acceptable rates of SSO, SSOPI, SSI, and recurrence.

## 1. Introduction 

Patients with incisional ventral hernias (VH) seek treatment to address functional issues like breathing difficulties, problems with bowel movements, urination, posture, and cosmetic concerns. Hernia repair should prioritize restoring the integrity of the abdominal wall while preserving the abdominal muscles.

Ventral hernias (VH) present significant challenges for surgeons due to the risk of recurrence, difficulties in aligning the abdominal muscles, and issues in determining the optimal layer for mesh placement. While the use of mesh in hernia repair is widely accepted, the primary concern revolves around identifying the most beneficial layer within the abdominal compartment [1,2]. 

Placing the mesh above the visceral area of the abdomen can lead to various complications, including bowel adhesions and intestinal fistulas. Bridging mesh techniques may result in anterior bulging and may compromise the strength of the abdominal wall. Onlay mesh placement carries the risks of seroma formation and surgical site complications due to extensive subcutaneous dissection [2,3]. However, aligning the fascial sides poses a major challenge for surgeons. 

This study aims to highlight the effectiveness of utilizing the anterior rectus fascia as a turnover flap and performing onlay mesh reinforcement, a procedure known as the modified Chevrel technique (MCT). This technique offers surgeons a viable approach with acceptable complication rates, potentially serving as a reliable solution amidst the complexities of VH repair. 

## 2. Materials and Methods 

We conducted a retrospective study involving patients who underwent MCT for abdominal hernias between January 2013 and December 2019. Data collection was carried out using patients’ records from our hospital’s archive and electronic database. Preoperatively, all patients or their relatives provided written informed consent. The interventions performed in this study adhered to the ethical standards outlined in the 1964 Helsinki Declaration and its subsequent amendments. All surgeries were performed by the same surgical team at a single institution. Ethical approval for our study was obtained from the Clinical Researchers’ Ethical Committee of Muğla Sıtkı Koçman University, Faculty of Medicine, with approval number 22/I, dated 27 December 2018.

Patients with missing data were excluded from the analysis. Demographic information, including age, sex, hernia etiology (primary/incisional), and body mass index (BMI) in kg/m^2^, was documented. Comorbidities such as hypertension, chronic obstructive pulmonary disease (COPD), and diabetes mellitus were also recorded. Additionally, data on the operative time, length of hospital stay, and timing of surveillance were collected from patient records. Postoperative complications were classified using the Clavien–Dindo classification system [4]. Complication data were collected for up to 30 days after surgery, and, if the treatment for a complication extended beyond 30 days, data collection continued accordingly. 

The measurements of hernia size and content followed the recommendations of the European Hernia Society [5]. Surgical site infection (SSI) and surgical site occurrences requiring procedural intervention (SSOPI) were identified based on previously published manuscripts and guidelines from the Centers for Disease Control and Prevention (CDC) [6,7,8]. SSIs were categorized as cellulitis and superficial, deep, and organ space infections. Notably, there were no occurrences of deep or organ space infections among the SSIs in our series. Wound erythema, which was treated with antibiotics but did not necessitate the interventional opening of surgical incisions, was identified as wound cellulitis. Sterile fluid collections (hematoma or seroma), wound dehiscence, or the identification of an enterocutaneous fistula were classified as SSOs. Procedural interventions such as percutaneous drainage, bedside wound exploration, or reoperation were described as SSOPI. The primary outcome regarding SSI, SSO, and SSOPI was recurrence. To assess the severity of a hernia, the modified Ventral Hernia Working Group grading scale (mVHWGs) was utilized [9]. 

Recurrence was assessed through intermittent physical examinations during outpatient follow-ups. In cases where clinical suspicion arose, recurrence was confirmed through abdominal computed tomography (CT). 

### 2.1. Surgical Technique

**Figure 1:**
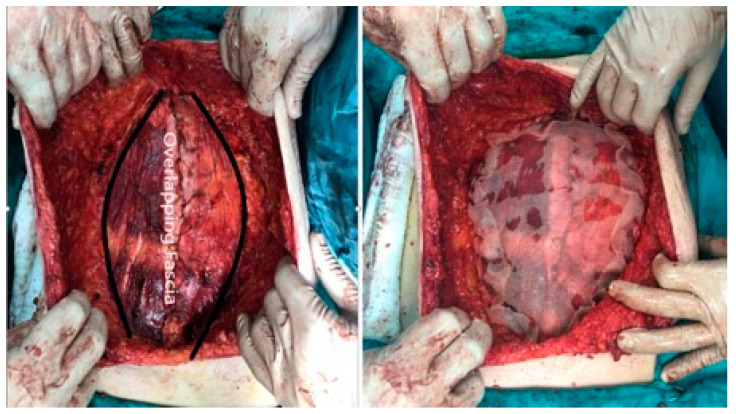
Illustration depicting the overlapping of the rectus abdominis anterior fascia and onlay mesh replacement.

**Figure 2:**
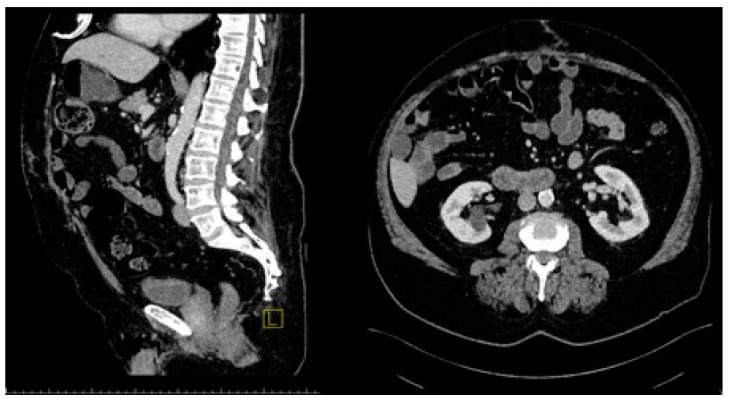
Computed tomography (CT) images, with sagittal and vertical views showing the mesh and overlapped fascia without bulging. (L on the image: left side).

The operative technique employed in our study follows detailed protocols outlined in previous manuscripts [10]. The intravenous administration of prophylactic antibiotics was standard practice, with reapplication if the duration of the operation exceeded 3 h. Regarding operative principles, a key modification from the original Chevrel technique involves reconstructing the linea alba. After subcutaneously delineating the hernia defect and conducting thorough adhesiolysis, the hernia defect in the midline is closed using both the original posterior rectus sheet and an overlapping anterior rectus sheet, as in the original Chevrel technique [11]. In the original technique, overlapping layers can lead to abdominal hypertension and challenges in closing the hernia gap. To address this, a modified approach is proposed, utilizing the anterior rectus sheath in a method akin to an open-book technique. The anterior rectus sheath is incised 2–5 cm from the midline, tailored to the hernia defect’s size. It is then separated from the rectus abdominis muscle fibers and placed over the hernia gap. The overlapping rectus sheath is sutured in a continuous fashion using 2/0 polypropylene sutures. During suturing, unintended gaps may arise in the fascial flaps’ merger, requiring additional primer sutures using the same suture material. This technique promotes more fascial flap availability, facilitating better abdominal wall reconstruction without relying solely on the linea alba. Additionally, mesh reinforcement is applied as a second layer, typically employing macroporous polypropylene mesh for all patients. Fixation initiated from the cranial and caudal edges of the defect ensures the proper orientation of mesh suturing (Figure 1). When applying the mesh onto the overlapped fascial flap, it is crucial to achieve tension-free reinforcement. This is achieved by securing the mesh to the incised site of the rectus abdominis with running sutures, ensuring that approximately 1 cm of excess mesh remains during tailoring. To manage drainage effectively, one or two vacuum drains are placed, based on the size of the subcutaneous dissection. Closure of the subcutaneous fat tissue is conducted using absorbable sutures, while any ischemic skin tissue is excised and primarily sutured separately. Post-operative CT images reveal no abdominal bulging, which can be attributed to the medialization of the myofascial flap (Figure 2). The drains are typically removed once the seroma generation is less than 50 mL, which is typically around 5 days post-surgery. If this threshold is not met within 10 days, the drain is still removed to mitigate the risk of ascending infection. Epidural anesthesia is not routinely administered. Patients are encouraged to mobilize from the first postoperative day and must wear a snug abdominal binder for at least 2 weeks, ensuring that the binder does not impede respiration to prevent seroma formation. Patients are advised to gradually resume normal activities, including heavy lifting, within 6 to 8 weeks post-surgery.

### 2.2. Statistical Analysis

The data analysis was conducted using SPSS statistics 22.0 software. Categorical variables were presented as counts, frequencies, and percentages, while continuous variables were described using means and standard deviations or medians. Demographic features and recurrence rates were examined using descriptive statistics. Categorical variables were assessed using Pearson’s chi-square or Fisher’s exact test, while continuous variables were analyzed using the independent-sample *t*-test for normally distributed data and the Mann–Whitney U test for non-normally distributed data. The level of statistical significance (α) was set at 0.05.

## 3. Results 

Between 2013 and 2019, a total of 321 ventral hernia (VH) patients underwent surgery, with 76 of them undergoing the modified Chevrel technique performed by the same surgical team. One patient unfortunately passed away, due to myocardial infarction on the first postoperative day, and was subsequently excluded from the study. The remaining patients underwent various other techniques, including laparoscopic sublay, open sublay, anterior compartment separation techniques, and transversus abdominis release. The mean age of the patients was 59.86 years, ranging from 32 to 93. Among male patients, the mean age was 59.7 years (ranging from 33 to 93), while among female patients, it was 59.9 years (ranging from 32 to 79). The body mass index (BMI) of the patients ranged from 19.2 to 37, with a mean of 29.1. The mean width of the hernia was 9.03 units. The mean operative time and length of hospital stay were 99.73 min and 6.88 days, respectively. Seven patients (11%) underwent concomitant procedures, six of which were cholecystectomies, and one was an oophorectomy. The oophorectomy was performed due to suspected malignancy, but the frozen sectional study revealed benign morphological disorders.

In Table 1, various parameters potentially contributing to SSI were assessed. Statistical analysis revealed no significant associations between BMI, hernia width, age, ASA score, and sex with SSI occurrence. However, there was a statistically significant association between the modified Ventral Hernia Working Group (VHWG) grade scale and SSI occurrence (*p* = 0.001), as illustrated in Table 2.

### Recurrence

Recurrence determination relies on suspected clinical findings and concurrent examination results, such as a hernia sac or fascial defect palpation, and is confirmed by a CT scan. The median follow-up period for patients is 42.9 months (ranging from 14 to 96), with a recurrence rate of 4% (3 cases). A statistically significant relationship was found between recurrence and the presence of COPD (*p* = 0.002). However, other comorbidities such as hypertension and diabetes were not found to be significantly related to recurrence. Additionally, all patients with recurrence were obese, although this association did not reach statistical significance (*p* > 0.05). The mean length of hospital stay for patients with recurrence was 17.6 days (ranging from 6 to 29). Two of the patients with recurrence experienced no wound complications and were discharged uneventfully after hernia surgery. However, upon recurrence detection, two patients declined further operations.

**Figure 3:**
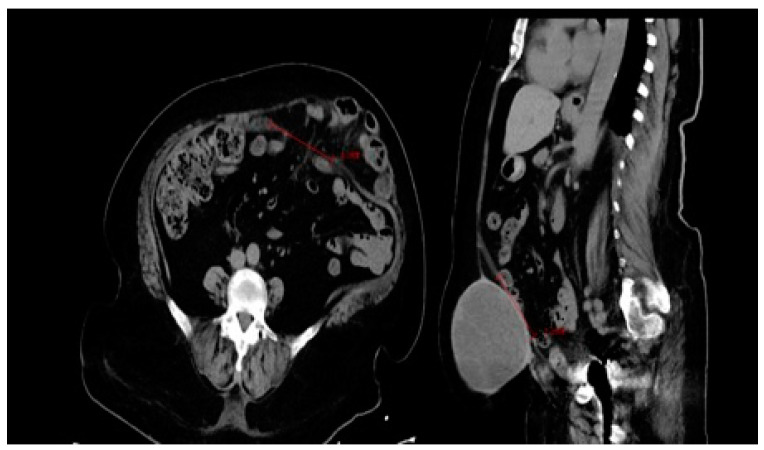
CT image revealing recurrence and the presence of a granuloma.

**Figure 4:**
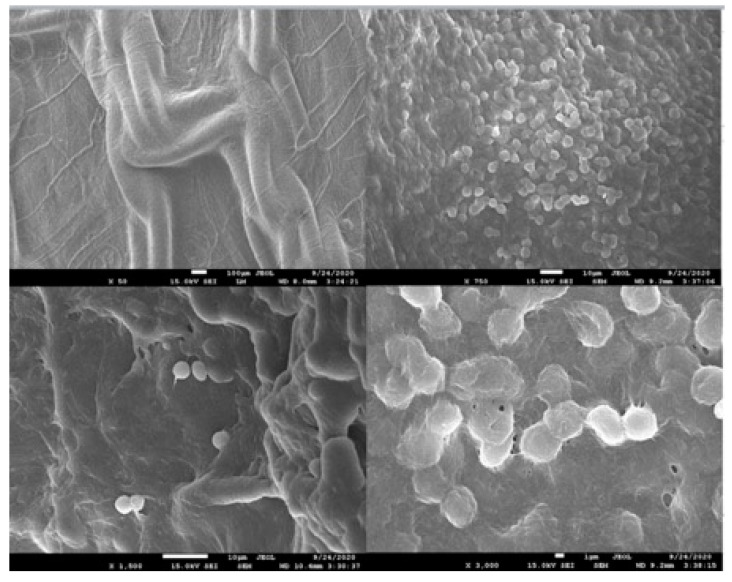
Original electron microscopic image displaying the mesh when resected for wound infection and granuloma.

The third recurrent patient experienced a deep wound infection extending to the mesh, necessitating treatment with negative-pressure wound therapy (NPWT) for two weeks. Their hospital stay lasted 29 days, and they had a BMI of 36 kg/m^2^. This patient returned to the hospital for reoperation 24 months after the initial surgery. During the initial evaluation, a 10 cm × 15 cm semisolid mass formation was observed in the operation area in the computed tomography scan (Figure 2). This mass was identified as meshoma-like granulation tissue formed during the surgical procedure, and histopathological evaluation confirmed this interpretation. Scanning electron microscopy examination of the mesh revealed fungal budding in the mesh area and severe breaks in the patch tissue, likely due to the deep wound infection and prolonged negative pressure wound therapy (Figure 3 and Figure 4). The patient underwent an anterior component separation technique for hernia repair and was discharged uneventfully on the fifth postoperative day.

It is worth noting that the hernia defects of the patients exhibiting recurrence were all below 10 cm in size during the initial surgery, although this finding did not reach statistical significance (*p* = 0.647).

## 4. Discussion

ACS and TAR are technically more difficult to apply than MCT. Release of the transversus abdominis muscle and separation of the external oblique muscle may disrupt the cylindrical structure of the abdomen. In MCT, the hernia defect is closed only with fascial flaps, without disrupting the structure of the muscle compartments. After the application of MCT, the other two techniques can be kept as reserve techniques and applied surgically in case of recurrence. 

In this study, we aimed to present our experiences with MCT and assess the factors influencing SSO and recurrence. We observed SSO, SSI, and SSOPI rates of 39 (52%), 12 (16%), and 32 (42%), respectively. Comparative data from recent meta-analyses showed varying rates of SSI and SSO in patients undergoing different techniques. For instance, open and robotic transversus abdominis release (TAR) treatment had SSI rates of 5.2% and 3.6%, and SSO rates of 11.5% and 5.3%, respectively [12]. Another meta-analysis reported SSO, SSI, and SSOPI rates of 21.7%, 9.13%, and 9.82%, respectively [13]. Evaluating anterior component separation (ACS) as an onlay hernia repair technique, another recent study found SSI, SSO, and SSOPI rates of 22.7%, 38%, and 27.3%, respectively [14]. While wound complications are inevitable, minimizing them remains a paramount surgical objective. Our study’s SSO, SSI, and SSOPI rates, though relatively high compared to open and robotic TAR, were comparable to those observed with ACS. The modified VHWG grading scale proves to be a highly practical tool for predicting the development of SSI in patients [9]. Notably, the modified VHWG grading scale emerged as a significant predictor of SSI development in our study (*p* < 0.05). Utilizing this scale enables proactive measures in high-risk patients, potentially mitigating postoperative complications.

Several manuscripts and meta-analyses have investigated MCT [10,15,16,17]. Essentially, MCT involves an onlay mesh replacement procedure utilizing a medially advanced myofascial flap. This approach offers undeniable benefits for closing hernia gaps in an autograft fashion. In emergency settings, where mechanical obstruction and abdominal hypertension pose critical challenges, closing the hernia gap becomes a paramount concern for surgeons. Some ventral hernia (VH) experts prefer a stepped procedure for treating incarcerated or strangulated hernia patients [18]. However, the modified Chevrel technique allows for the advancement of myofascial flaps medially, facilitating the closure of the gap in a single procedure. It is worth noting that emergency hernia repair carries significantly higher mortality rates and often necessitates more bowel resections compared to elective repairs [19]. Nonetheless, in our study, 11 patients (14.8%) underwent surgery in an emergency setting without experiencing recurrences during the follow-up period. Numerous techniques have been described for incisional hernias, but MCT stands out as a versatile approach that is suitable for emergency, elective, clean, and clean-contaminated surgical sites [16].

Seroma formation is a natural consequence of the onlay process involved in the Modified Chevrel Technique, primarily due to subcutaneous dissection. In our experience, seroma and hematoma formation occurred in up to 17 patients (29.4%), with 15 patients (20%) requiring bedside wound care and aspiration, despite the use of negative pressure drains. Notably, if the subcutaneous dissection extends beyond 2 cm, seroma formation becomes more likely [20]. 

Patients often seek surgical intervention for VHs, primarily due to abdominal bulging, which may lead to aesthetic concerns and impairments in micturition, defecation, and respiratory functions [21,22]. The deterioration of the cylindrical structure of the trunk, abdomen, and diaphragm can hinder patients from effectively increasing abdominal pressure. Smaller hernias may exacerbate certain symptoms, such as intermittent abdominal pain due to incarceration.

The restoration of the linea alba and medialization of the rectus abdominis muscles are crucial for returning abdominal functions to normal. Without these corrections, patients may continue to experience abdominal wall bulging and observe the medialization of the linea alba and rectus abdominis. Based on our experience, we have found that the retraction of the mesh placed on the edges of the rectus sheath contributes to the reconstruction of the linea alba. 

Postoperative bulging is a distressing occurrence for patients that is often described as a bulge in the operative area, which can be challenging to differentiate from a recurrent hernia [23]. Nomenclature varies among authors, with terms such as “mesh protrusion”, “eventration”, and “pseudorecurrence” being used interchangeably [23,24]. In cases where mesh reinforcement is bridged, bulging becomes a significant concern [25]. Techniques such as anterior compartment separation (ACS) and lateral transversus abdominis release (TAR) may potentially lead to long-term lateral bulging. It has been established that ACS can induce external oblique atrophy, while TAR can induce transversus abdominis atrophy. However, in the modified Chevrel technique (MCT), there is no muscle separation involved, thus preserving the initial abdominal muscle anatomy to a greater extent. Consequently, if a recurrent hernia is encountered, the operator can utilize separation techniques such as TAR and ACS, as we did for our patients with recurrence.

Hernia recurrence stands as the most significant outcome of these surgical interventions, prompting a need to understand its etiology and implement preventive measures. In our cohort, recurrence occurred in three patients, all of whom were diagnosed with COPD, a statistically significant finding. Additionally, these patients had prolonged hospital stays, underwent NPWT, and were obese. Notably, the hernia defect size was below 10 cm. Recent meta-analyses have identified different cutoff points for hernia defect size, with a hernia defect below 10 cm having an odds ratio of 2.13 and a defect size of 15 cm having an odds ratio of 2.33 for recurrence [26]. Interestingly, COPD was found to have an odds ratio of 1.53 for recurrence in the same study. While this finding may seem contradictory to the existing literature, it suggests that COPD may have a more significant impact on hernia recurrence compared to hernia size in our patient population.

Prolonged NPWT lasting two weeks due to infection may lead to biofilm activity and fungal budding on the mesh. This underscores the importance of recognizing the potential risks associated with extended NPWT and wound infections, which should serve as warning signs for recurrence and meshoma development [27]. Extensive subcutaneous dissection can contribute to seroma formation, and if the perfusion of the flap is compromised, it can further facilitate the development of wound infection. Therefore, preserving the perforating vessels of the subcutaneous dissection and fascial flap is crucial to minimize the risk of requiring NPWT.

Our study is a retrospective study, and the number of patients is relatively small. Retrospective studies have limitations and, of course, randomized controlled studies are needed to determine the most ideal technique.

## 5. Conclusions

In conclusion, VH repair poses significant challenges for surgeons, as achieving effective abdominal closure is essential for both physiological function and aesthetic outcome. In this context, MCT offers a relatively simpler alternative to abdominal closure techniques like TAR and ACS. Despite its simplicity, MCT yields acceptable rates of SSO, SSOPI, SSI, and recurrence.

## Figures and Tables

**Table 1:** Demographics and baseline characteristics associated with surgical site infections (SSI).

		SSI				
		None N (%)	Cellulitis n (%)	Superficial n (%)	Deep n (%)	*p*-Value
Hernia width (cm)	<10	43 (57.3)	5 (6.6)	2 (2.6)	1 (1.3)	0.173
	≥10	20 (26.6)	1 (1.3)	0 (0)	3 (3.9)	
BMI	<30	37 (49.3)	5 (6.6)	1 (1.3)	1 (1.3)	0.329
	≥31	26 (24.6)	1 (1.3)	1 (1.3)	3 (3.9)	
Age	<65	37 (49.3)	5 (6.6)	1 (1.3)	2 (2.6)	0.647
	≥65	26 (24.6)	1 (1.3)	1 (1.3)	2 (2.6)	
ASA	I–II	41 (54.6)	5 (6.6)	0 (0)	1 (1.3)	0.71
	III–IV	22 (29.3)	1 (1.3)	2 (1.3)	3 (3.9)	
Sex	Male	20 (26.6)	3 (3.9)	1 (1.3)	1 (1.3)	0.754
	Female	43 (57.3)	3 (3.9)	1 (1.3)	3 (3.9)	
Modified VHWG	grade 1	36 (48)	5 (6.6)	0 (0)	1 (1.3)	0.001
	grade 2	25 (33.3)	1 (1.3)	1 (1.3)	1 (1.3)	
	grade 3	2 (2.6)	0 (0)	1 (1.3)	2 (2.6)	

**Table 2:** Perioperative characteristics of the patients.

	Frequency	Percent (%)
ASA		
1	11	14.7
2	36	48
3	25	33.3
4	3	4
Modified VHWG		
Grade 1	42	56
Grade 2	28	37.3
Grade 3	5	6.7
EHS classification		
M2 epigastric	10	13.3
M3 periumbilical	52	69.3
M4 infraumbilical	13	17.3
Hernia content		
Omentum	27	36
Small intestines	34	45.3
Colon	14	18.7
SSI		
None	63	84
Cellulitis	6	8
Superficial	2	2.7
Deep/Peritoneal	4	5.3
SSO		
None	36	48
Sterile fluid collection/seroma	17	22.7
Sterile fluid collection/hematoma	5	6.7
Dehiscence	6	8
Cellulitis	5	6.7
Surgical site infection/superficial	2	2.7
Surgical site infection/deep	4	5.3
SSOPI/Treatment		
None	51	68
Bedside wound care/aspiration	15	20
Debridement and suturing	5	6.7
Negative pressure wound therapy	4	5.3
Clavien–Dindo classification		
No complication	39	52
Grade 1	5	6.7
Grade 2	17	22.7
Grade 3a	7	9.3
Grade 3b	6	8
Grade 4	1	1.3
BMI kg/m^2^		
>30	42	56
<30	33	44
Comorbidities		
None	45	60
Diabetes	13	17.3
Hypertension	4	5.3
COPD	13	17.3
Operative setting		
Elective	64	85.1
Emergency	11	14.8

## Data Availability

No data is available due to privacy or ethical restrictions.
